# A new model based on gamma-glutamyl transpeptidase to lymphocyte ratio and systemic immune-inflammation index can effectively predict the recurrence of hepatocellular carcinoma after liver transplantation

**DOI:** 10.3389/fonc.2023.1178123

**Published:** 2023-04-20

**Authors:** Weiqi Zhang, Yi Bi, Kai Yang, Yan Xie, Zhaoxian Li, Xinghui Yu, Li Zhang, Wentao Jiang

**Affiliations:** ^1^School of Medicine, Nankai University, Tianjin, China; ^2^Department of Liver Transplantation, Tianjin Medical University First Center Clinical College, Tianjin, China; ^3^Department of Liver Transplantation, Tianjin First Center Hospital, Tianjin, China; ^4^Laboratory of Molecular and Treatment of Liver Cancer, Tianjin First Center Hospital, Tianjin, China

**Keywords:** hepatocellular carcinoma, liver transplantation, recurrence, prediction, prognosis, gamma-glutamyl transpeptidase-to-lymphocyte ratio, systemic immune-inflammation index

## Abstract

**Background:**

Liver transplantation (LT) is one of the most effective treatment modalities for hepatocellular carcinoma (HCC), but patients with HCC recurrence after LT always have poor prognosis. This study aimed to evaluate the predictive value of the gamma-glutamyl transpeptidase-to-lymphocyte ratio (GLR) and systemic immune-inflammation index (SII) in terms of HCC recurrence after LT, based on which we developed a more effective predictive model.

**Methods:**

The clinical data of 325 HCC patients who had undergone LT were collected and analyzed retrospectively. The patients were randomly divided into a development cohort (n = 215) and a validation cohort (n = 110). Cox regression analysis was used to screen the independent risk factors affecting postoperative recurrence in the development cohort, and a predictive model was established based on the results of the multivariate analysis. The predictive values of GLR, SII and the model were evaluated by receiver operating characteristic (ROC) curve analysis, which determined the cut-off value for indicating patients’ risk levels. The Kaplan-Meier survival analysis and the competing-risk regression analysis were used to evaluate the predictive performance of the model, and the effectiveness of the model was verified further in the validation cohort.

**Results:**

The recurrence-free survival of HCC patients after LT with high GLR and SII was significantly worse than that of patients with low GLR and SII (P<0.001). Multivariate Cox regression analysis identified GLR (HR:3.405; 95%CI:1.954-5.936; P<0.001), SII (HR: 2.285; 95%CI: 1.304-4.003; P=0.004), tumor number (HR:2.368; 95%CI:1.305-4.298; P=0.005), maximum tumor diameter (HR:1.906; 95%CI:1.121-3.242; P=0.017), alpha-fetoprotein level (HR:2.492; 95%CI:1.418-4.380; P=0.002) as independent risk factors for HCC recurrence after LT. The predictive model based on these risk factors had a good predictive performance in both the development and validation cohorts (area under the ROC curve=0.800, 0.791, respectively), and the performance of the new model was significantly better than that of single GLR and SII calculations (P<0.001). Survival analysis and competing-risk regression analysis showed that the predictive model could distinguish patients with varying levels of recurrence risk in both the development and validation cohorts.

**Conclusions:**

The GLR and SII are effective indicators for evaluating HCC recurrence after LT. The predictive model based on these indicators can accurately predict HCC recurrence after LT and is expected to guide preoperative patient selection and postoperative follow-up.

## Introduction

Hepatocellular carcinoma (HCC) is among the most common malignant tumors and the third leading cause of cancer-related death (1). Although there are many treatment modalities for HCC, such as hepatectomy, transarterial chemoembolization, radiotherapy, and targeted therapy, the prognosis of patients with HCC is still not satisfactory (2). In China, HCC is often associated with hepatitis B virus (HBV) infection and HBV-related liver cirrhosis (3). Importantly, the above-mentioned tumor treatments modalities cannot solve the problem of liver cirrhosis. As one of the most effective treatment modalities, liver transplantation (LT) not only removes the tumor completely but also eliminates liver cirrhosis (4). For patients who meet the Milan criteria, the 5-year survival rate after LT is up to 70% (5, 6). However, some patients experience tumor recurrence after LT, which seriously affects patients’ prognosis.

There are some recognized criteria for selecting patients with HCC before LT to optimize prognosis, such as the Milan criteria (5) and the University of California, San Francisco criteria (7). However, these criteria either strictly exclude some patients who can benefit from LT or do not accurately identify high-risk groups, so additional indicators are needed to assess prognosis. Although pathological features of HCC, such as tumor differentiation and microvascular invasion, are important and accurate prognostic factors, it is not easy to obtain the information preoperatively (8). Previous studies have shown that the systemic inflammatory response can promote angiogenesis, DNA damage, and tumor invasion by upregulating cytokine proliferation (9–11). The relationship between inflammation, the occurrence and progression of HCC, and prognosis is increasingly accepted. The gamma-glutamyl transpeptidase-to-lymphocyte ratio (GLR) can be used to predict HCC recurrence and patients’ prognosis after hepatectomy (12, 13). The systemic immune-inflammation index (SII), calculated as platelet count × neutrophil-to-lymphocyte ratio, has also been shown to be associated with HCC recurrence prediction (14, 15). Few studies have investigated the value of these indicators in terms of HCC recurrence prediction after LT. This study aimed to explore the value of both the GLR and SII in predicting HCC recurrence and to establish a predictive model for HCC recurrence after LT based on preoperative indices.

## Materials and methods

### Patients

The preoperative and postoperative clinicopathological data of HCC patients who received LT at the Tianjin First Central Hospital between July 2014 and June 2019 was collected retrospectively. The inclusion criteria were as follows (1): the diagnosis of HCC was confirmed by histopathological evaluation;(2) received orthotopic LT;(3) no macrovascular invasion and distant metastasis. And the exclusion criteria were as follows: (1) preoperative complication of other tumors or having history of malignancies; (2) combined multi-organ transplantation; (3) incomplete clinical and follow‐up data; (4) preexisting autoimmune or systemic inflammatory disease or diseases of the hematologic system. A total of 325 patients were included in this study. These patients were randomly divided into a development cohort (n = 215) and a validation cohort (n = 110) at a ratio of 2:1. All study procedures were performed in accordance with the ethical guidelines of the Helsinki Declaration and approved by the Tianjin First Central Hospital clinical research ethics committee.

### Surgery and postoperative management

All patients received ABO-compatible deceased-donor livers. Postoperatively, recipients were prescribed a triple immunosuppressive regimen consisting of tacrolimus, mycophenolate mofetil, and methylprednisolone. Basiliximab (20 mg) was intravenously infused intraoperatively and 4th day after LT.

### Definitions

Recurrence-free survival (RFS) time was defined as the period from LT to tumor recurrence, and for patients who were lost to follow-up or died without experiencing tumor recurrence, the RFS time was the period from LT to the last follow-up. Overall survival (OS) time was defined as the period from LT to death or the last follow-up.

### Follow up

Follow-up was carried out in outpatient clinic or *via* telephone interviews. The patients were followed up every 2 months within 2 years and every 6 months thereafter. The routine imaging and laboratory examinations were performed during the follow-up period. When the metastasis or recurrence was suspected, it was further evaluated by the magnetic resonance imaging or the enhanced computed tomography. The deadline for follow-up was December 31st, 2022.

### Statistical analysis

Statistical analysis was performed using SPSS Statistics for Windows, version 26.0 (IBM Corp). The measurement data were presented as $\bar{x}\pm SD$ or M (P25 ~ P75), and the t-test or Mann-Whitney U test was used for intergroup comparisons. Categorical data were presented as frequency and percentage, and the χ^2^ test was used for intergroup comparisons. Survival curves were constructed using the Kaplan-Meier method, and the differences between curves were evaluated using the log-rank test. Cox proportional hazards regression analysis (the forward stepwise method) was used for univariate and multivariate analysis, and the significant factors from the univariate analysis (P<0.1) were incorporated into multivariate analysis to determine the independent risk factors affecting recurrence. A scoring model was established according to the results of the Cox proportional hazards regression analysis. Patients were given scores according to the β coefficient of each index. For each patient, the individual risk factor score was summed to determine a comprehensive risk score. The threshold of risk index for predicting tumor recurrence was determined by the maximum Youden index in the receiver operating characteristic (ROC) curve. Overall predictive performance was indicated by the area under the ROC curve (AUC). The R (version 4.1.0) software was used to perform competing-risk regression analysis to assess the utility of the model with death acting as a competing risk to recurrence. The main R packages used included “foreign,” and “cmprsk.” All P values were two-tailed, and P<0.05 indicated statistical significance.

## Results

### Patient characteristics

**Figure 1:**
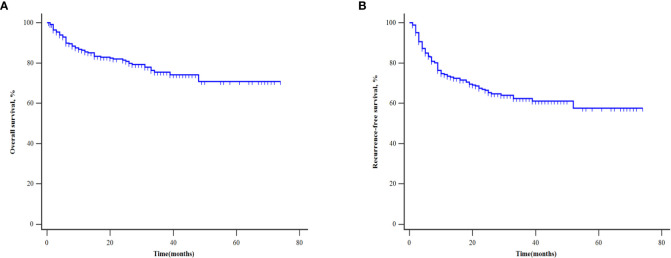
Kaplan‐Meier curves of patients for overall survival **(A)** and recurrence-free survival **(B)**.

The general data of patients are shown in **Table 1**. Among the 325 patients, there were 288 males (88.62%) and 37 females (11.38%), with a mean age of 54.47 ± 9.32 years. HBV infection was the most common cause of HCC, accounting for 72.92% of the diagnoses. The median follow-up time was 30.00 months. During follow-up, 60 patients died, and 93 patients experienced tumor recurrence. The 3-year and 5-year OS rates were 75.45% and 70.83%, respectively (**Figure 1A**). And the 3-year and 5-year RFS rates were 62.41% and 57.49%, respectively (**Figure 1B**). There were no significant difference between the development and validation cohorts in terms of any of the investigated variables (**Table 1**).

**Table 1:** The baseline data of patients in the development and validation cohorts.

Characteristics	Development cohort (n=215)	Validation cohort (n=110)	P value
Age(years)	54.57 ± 9.24	54.26 ± 9.52	0.778
Gender			0.199
Male	194(90.2)	94(85.5)	
Female	21(9.8)	16(14.5)	
Etiology			0.101
HBV	163(75.81)	74(67.27)	
Non-HBV	52(24.19)	36(32.73)	
Cirrhosis			0.427
Yes	187(86.98)	99(90.00)	
No	28(13.02)	11(10.00)	
Tumor number			0.872
Multiple	127(59.1)	66(60.0)	
Single	88(40.9)	44(40.0)	
Maximum tumor diameter(cm)	3.00(2.00,5.50)	3.00(2.00,6.00)	0.867
Satellite nodules			0.233
Yes	25(11.6)	18(16.4)	
No	190(88.4)	92(83.6)	
AFP(μg/L)	40.74(4.84,747.80)	37.37(6.34,563.00)	0.907
TBIL(μmol/L)	24.51(14.83,51.45)	20.82(13.28,47.85)	0.157
AST(U/L)	43.90(29.20,73.60)	40.90(28.58,77.35)	0.758
ALT(U/L)	34.70(21.40,52.90)	32.10(21.40,49.48)	0.431
ALB(g/L)	36.26 ± 6.23	36.93 ± 5.72	0.351
CER(μmol/L)	69.00(59.00,81.00)	66.98(53.00,76.05)	0.126
INR	1.23(1.09,1.42)	1.23(1.08,1.37)	0.415
MELD score	8.31(7.03,10.17)	7.86(6.95,9.97)	0.331
GLR	101.83(49.55,224.88)	103.82(52.87,232.22)	0.702
SII	279.93(129.74,650.00)	298.29(146.16,591.10)	0.473

HBV, hepatitis B virus; AFP, alpha-fetoprotein; TBIL, total bilirubin; AST, aspartate aminotransferase; ALT, alanine aminotransferase; ALB, albumin; CER, creatinine; INR, international normalized ratio; MELD, model for end-stage liver disease; GLR, gamma-glutamyl transpeptidase to lymphocyte ratio; SII, systemic immune-inflammation index.

### The predictive value and cut-off value of each index in predicting postoperative tumor recurrence in patients with HCC

**Figure 2:**
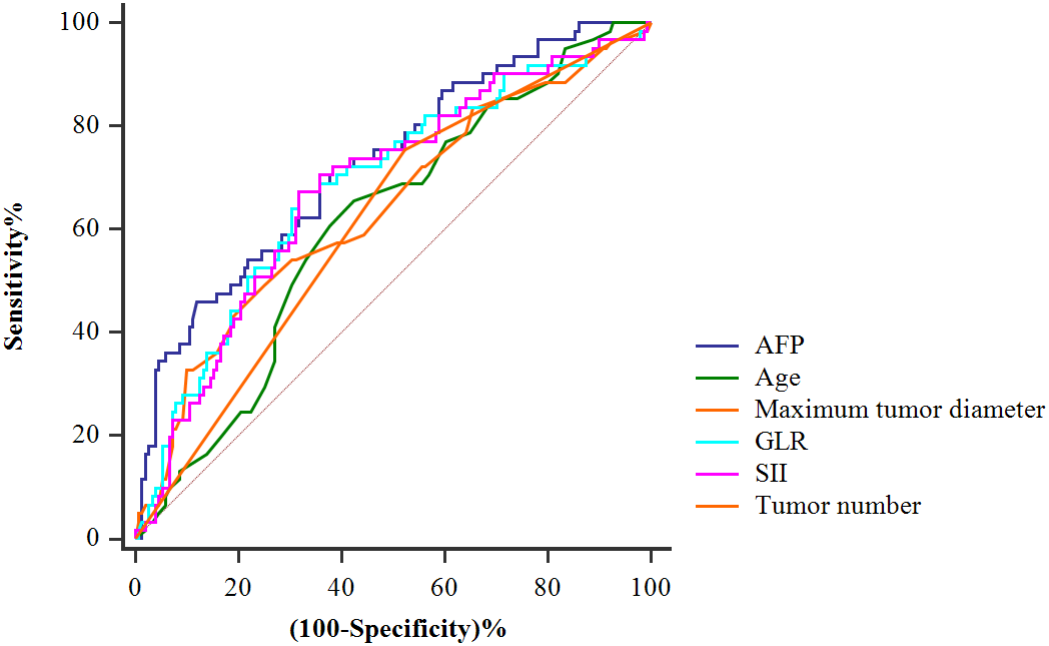
Receiver-operating characteristic curves for tumor recurrence of the AFP, age, maximum tumor diameter, tumor number, GLR, and SII.

**Figure 2** shows the predictive value of each index for HCC recurrence after LT, as determined by ROC curve analysis. Alpha-fetoprotein (AFP) level, GLR, and SII were stronger predictors of HCC recurrence than the age, maximum tumor diameter and tumor number. According to Youden index analysis, the best cut-off values for age, maximum tumor diameter, tumor number, AFP level, GLR, and SII were 55.00, 5.15, 1, 55.50, 133.98, and 334.46 respectively (**Table 2**).

**Table 2:** The area under curve and cut-off value of variables.

Variables	AUC (95% CI)	P value	Cut-off value	Sensitivity (%)	Specificity (%)
Age (years)	0.608 (0.528-0.689)	0.013	55.00	64.5	57.5
Maximum tumor diameter (cm)	0.629 (0.545-0.713)	0.003	5.15	41.9	80.4
AFP (μg/L)	0.724 (0.649-0.800)	<0.001	55.50	68.9	63.8
Tumor number	0.618 (0.549-0.683)	0.004	1	75.81	47.71
GLR	0.686 (0.606-0.766)	<0.001	133.98	67.7	68.4
SII	0.683 (0.604-0.762)	<0.001	334.46	67.7	68.4

AFP, alpha-fetoprotein; GLR, gamma-glutamyl transpeptidase to lymphocyte ratio; SII, systemic immune-inflammation index; CI, confidence interval; AUC, area under curve.

### The effect of GLR and SII on the recurrence-free survival of patients

**Figure 3:**
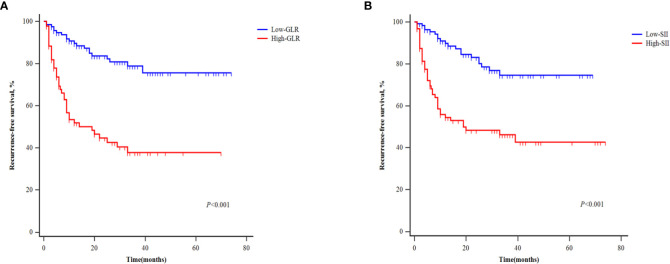
Kaplan‐Meier curves of patients with different levels of GLR **(A)** and SII **(B)** for recurrence-free survival.

Patients in the development cohort were divided into the high-GLR group and low-GLR group or the high-SII group and low-SII group, according to the respective cut-off values. Survival analysis showed that the postoperative RFS of patients with high GLR was significantly worse than that of patients with low GLR (P<0.001) (**Figure 3A**). Patients with high SII also had significantly worse RFS outcomes than patients with low SII (P<0.001) (**Figure 3B**).

### Univariate and multivariate analysis of postoperative RFS in patients with HCC

Univariate analysis showed that the tumor number (P<0.001), maximum tumor diameter (P<0.001), AFP level (P<0.001), GLR (P<0.001), and SII (P<0.001) were all significantly associated with RFS among HCC patients. Multivariate analysis indicated tumor number (HR:2.368; 95%CI:1.305-4.298; P=0.005), maximum tumor diameter (HR:1.906; 95%CI:1.121-3.242; P=0.017), AFP level (HR:2.492; 95%CI:1.418-4.380; P=0.002), GLR (HR:3.405; 95%CI:1.954-5.936; P<0.001), and SII (HR: 2.285; 95%CI: 1.304-4.003; P=0.004) as independent risk factors for HCC recurrence after LT (**Table 3**).

**Table 3:** Univariate and multivariate Cox regression analysis of risk factors for recurrence-free survival of HCC patients after LT.

Variables	Univariate Analysis		Multivariate Analysis	
	HR (95% CI)	P value	HR (95% CI)	P value
Age (>55 years)	0.613 (0.387-0.970)	0.180		
Gender (male)	0.851 (0.446-1.621)	0.453		
Etiology (HBV)	0.854 (0.493-1.479)	0.573		
Cirrhosis (Yes)	0.940 (0.463-1.905)	0.863		
Tumor number (multiple)	2.881 (1.609-5.160)	<0.001	2.368 (1.305-4.298)	0.005
Maximum tumor diameter (>5.15 cm)	2.996 (1.791-5.012)	<0.001	1.906 (1.121-3.242)	0.017
Satellite nodules (Yes)	1.126 (0.609-2.081)	0.361		
AFP (>55.50 μg/L)	3.475 (2.021-5.974)	<0.001	2.492 (1.418-4.380)	0.002
GLR (>133.98)	4.240 (2.480-7.249)	<0.001	3.405 (1.954-5.936)	<0.001
SII (>334.46)	3.495 (2.050-5.959)	<0.001	2.285 (1.304-4.003)	0.004

HBV, hepatitis B virus; AFP, alpha-fetoprotein; GLR, gamma-glutamyl transpeptidase to lymphocyte ratio; SII, systemic immune-inflammation index; HR, hazard ratio; CI, confidence interval.

### Development and evaluation of the predictive model

**Figure 4:**
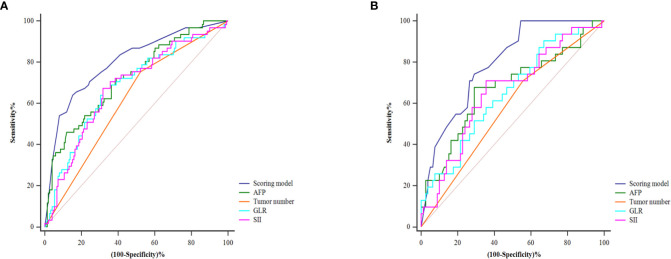
The predictive performance of the model and the comparison with the AFP, tumor number, GLR and SII in the development **(A)** and validation **(B)** cohorts.

**Figure 5:**
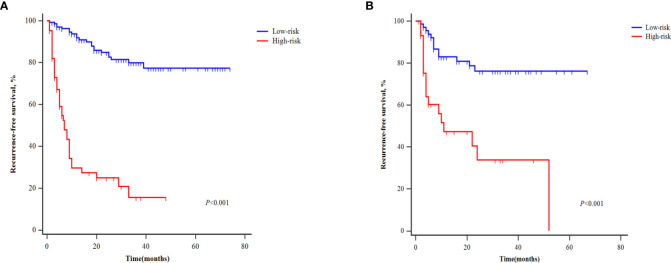
Kaplan‐Meier curves of patients with different recurrence risk for recurrence-free survival in the development **(A)** and validation cohorts **(B)**.

**Figure 6:**
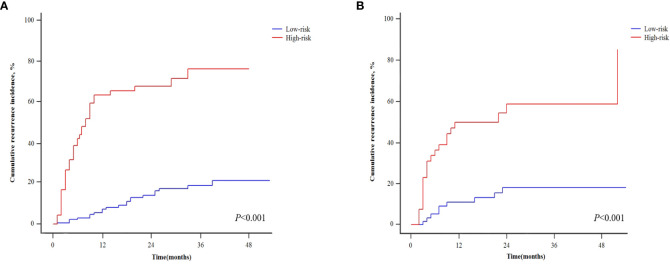
Competing-risk regressions for patients with different recurrence risk in the development **(A)** and validation cohorts **(B)**.

**Table 4** shows the patients’ risk scores according to the β coefficient of each index in the multivariate Cox regression analysis. The AUC of the model was 0.800 (95%CI: 0.732-0.867; P<0.001). Hanley-McNeil analysis showed that the scoring model was significantly better than the GLR, SII, tumor number and AFP in the development cohort (P=0.001, 0.001, <0.001, 0.038, respectively) (**Figure 4A**). The patients were divided into high-risk and low-risk groups according to a cut-off value of 27.00 with a sensitivity of 66.1% and specificity of 82.4%. Survival analysis showed that the model could well distinguish the patients with varying levels of recurrence risk (**Figure 5A**). As shown in **Figure 6A**, the competing-risk regression analysis revealed a statistically significant difference between the two groups (Gray’s test, P<0.001).

**Table 4:** Scores for independent risk factors of tumor recurrence.

Variables	β-coefficient	Score
Tumor number (multiple)	0.862	9
Maximum tumor diameter (>5.15 cm)	0.645	6
AFP (>55.50 μg/L)	0.913	9
GLR (>133.98)	1.225	12
SII (>334.46)	0.826	8

AFP, alpha-fetoprotein; GLR, gamma-glutamyl transpeptidase to lymphocyte ratio; SII, systemic immune-inflammation index.

### Validation of the predictive model

The predictive model also performed well in the validation cohort with the AUC was 0.791 (95%CI: 0.705-0.876; P<0.001). Hanley-McNeil analysis showed that the scoring model was also significantly better than the GLR, SII, tumor number and AFP in the validation cohort (P=0.006, 0.023, <0.001, 0.044, respectively) (**Figure 4B**), with a sensitivity of 71.0% and specificity of 72.2%. Survival analysis showed that the model could also distinguish patients with varying levels of recurrence risk in the validation cohort (**Figure 5B**). The competing-risk regression analysis further verified the predictive value of the model (Gray’s test, P<0.001) (**Figure 6B**).

## Discussion

Tumor recurrence is one of the most common causes of death in HCC patients after LT, which seriously affects the prognosis of patients (16–18). At present, the selection criteria of HCC for LT, such as Milan criteria, the University of California, San Francisco criteria, only incorporate tumor load, distant metastasis and macrovascular invasion. Even if the Milan criteria are met, there are still 15-20% of patients with postoperative tumor recurrence (19–21). Therefore, some additional indicators are needed to assess the prognosis of patients.

Some studies have shown that the immune inflammatory state is closely related to the progression of tumors. Increased neutrophil proliferation plays an important role in the occurrence and development of tumors. Neutrophils can induce tumor proliferation and angiogenesis to promote the migration and metastasis of tumor cells. Hepatoma cells can also induce neutrophils to release cell growth factors, leading cancer cells to be more aggressive (14, 22). Platelets can be activated by tumor cells and form tumor emboli through adhesion molecules on their surfaces to protect tumor cells from the killing effect of the immune system and promote tumor cell metastasis (23, 24). Additionally, platelets and neutrophils can secrete vascular endothelial growth factor (VEGF) to promote tumor progression (25). Lymphocytes play a role in anti-tumor immunity, which involves the secretion of a series of cytokines and the induction of cytotoxic cell death, thus inhibiting the proliferation and migration of tumor cells. Decreased circulation of lymphocytes in the peripheral blood weakens the body’s defenses, leading to tumor recurrence and progression (26). The SII, as a simple and easily calculable indicator, reflects the body’s immune and inflammatory state. It is also considered to be a powerful prognostic indicator of poor outcomes in patients with HCC or cholangiocarcinoma (27, 28). In this study, we also found that the RFS in patients with high SII was significantly lower than that in patients with low SII. The SII was an independent risk factor for HCC recurrence after LT.

Gamma-glutamyltransferase (GGT), as a cell surface enzyme, has been proven to be a marker of many cancers (29–31). Tumor invasion and the release of inflammatory factors often lead to the destruction of hepatocytes, which is characterized by increased GGT in the peripheral blood (32, 33). It has been reported that GGT is a reliable marker of oxidative stress, and the increased expression of GGT in tumor cells is associated with the production of ROS, which can promote the invasion and migration of tumor cells (34, 35). Therefore, there is a certain relationship between the GLR and HCC recurrence. Previous studies have shown that the GLR can be used as a potential indicator of early recurrence and prognosis of HCC (36, 37). Zhang et al. (38) also found that the GLR could be used as a predictor of HCC microvascular invasion and that high levels of GLR were associated with patients’ poor prognosis. However, few studies have investigated the relationship between the GLR and the prognosis of HCC patients after LT.

In this study, we found that the RFS of patients with high GLR was significantly lower than that of patients with low GLR and a high level of GLR was an independent risk factor of HCC recurrence after LT. Considering the predictive value of a single factor is limited, the predictive model combining the GLR and SII with tumor load and AFP level was established and demonstrated a good predictive value in both the development and validation cohorts. The predictive performance of the model was superior to those of the single indices. As the death is a competitive factor for tumor recurrence, we used competing-risk regression to correct this. We found that, in both the development and validation cohorts, the model could still well distinguish people with varying levels of tumor recurrence risk. In particular, the model only used preoperative imaging and serological indices, and it not only predicted tumor recurrence, but also potentially can be used to select patients suitable for LT.

This study had some limitations. Firstly, this was a single-center, retrospective study, so the model was only validated internally without externally generalizable. Secondly, the sample size was relatively small. Therefore, further larger-scale, multicenter, prospective validation studies are needed.

## Conclusion

Preoperative selection and postoperative follow-up of HCC patients undergoing LT are important elements of the management of this patient population. This study explored the value of the GLR and SII in predicting HCC recurrence after LT, and it established a simple and effective predictive model that could help to guide the pre-LT selection and post-LT follow-up of patients with HCC.

## Data availability statement

The raw data supporting the conclusions of this article will be made available by the authors, without undue reservation.

## Ethics statement

The studies involving human participants were reviewed and approved by Tianjin First Central Hospital clinical research ethics committee. Written informed consent for participation was not required for this study in accordance with the national legislation and the institutional requirements.

## Author contributions

WZ, YB, and KY were responsible for data analysis, drafting and revision of the manuscript. YX, ZL, and XY were responsible for data acquisition. LZ and WJ were responsible for study conception and design. All authors contributed to the article and approved the submitted version.
